# Perceptions of telehealth in real‐world oncological care: An exploration of matched patient‐ and clinician‐reported acceptability data from an Australian cancer centre

**DOI:** 10.1002/cam4.4700

**Published:** 2022-04-04

**Authors:** Anna Collins, Sue‐Anne McLachlan, Leeanne Pasanen, Olivia Wawryk, Jennifer Philip

**Affiliations:** ^1^ Department of Medicine, St Vincent′s Hospital Melbourne University of Melbourne Melbourne Victoria Australia; ^2^ Medical Oncology St Vincent′s Hospital Melbourne Melbourne Victoria Australia; ^3^ Parkville Integrated Palliative Care Service Peter MacCallum Cancer Centre Melbourne Victoria Australia

**Keywords:** care delivery, oncologists, patients, perceptions, prospective cross‐sectional survey, telehealth

## Abstract

**Background:**

Prior to 2020, the use of telehealth in cancer care was limited, but COVID‐19 necessitated its rapid and widespread adoption into routine care delivery. This study aimed to evaluate perceptions of telehealth through a dyadic exploration of matched cancer patient‐ and clinician‐reported acceptability data and to explore factors that may predict greater suitability for telehealth.

**Methods:**

A prospective, cross‐sectional, exploratory survey study assessed (matched) patient‐ and clinician‐reported perceptions of telehealth consultations occurring at a metropolitan, tertiary‐based cancer centre in Victoria, Australia.

**Results:**

One‐hundred and fifty‐five matched patient‐ and clinician‐reported data were included. High rates of acceptability with telehealth were reported by patients (93%) and clinicians (91%), who mostly shared concordant views (86%). Factors significantly associated with increased acceptability for telehealth, included, for clinicians, greater familiarity with the patient (OR 8.20, 95% CI: 1.50–45.06, *p* = 0.02), and younger patient age (OR 1.06, 95% CI: 0.99–1.13, *p* = 0.05), and for patients was earlier stage disease (≤stage III) (OR 5.29, 95% CI: 1.08–25.82, *p* = 0.04). Lower acceptability for telehealth according to clinicians was associated with poorer patient performance status (OR 0.04, 95% CI 1.00–1.08, *p* = 0.04) and for patients with the need for an interpreter (0R 0.06, 95% CI: 0.008–0.51, *p* = 0.009).

**Conclusion:**

While overall telehealth is acceptable in cancer care, our findings raise important implications for future service development, notably that it may be less optimal for patients with higher complexity of need—including those with more advanced disease, poorer performance status, those less well known to treating clinicians and those identified to have additional language barriers.

## Graphical abstract

This matched survey of cancer patient and clinician views of telehealth as they reflected on the same recent consultation showed both groups found it high acceptable. However lower ratings of acceptability were associated with less familiarity between patient and clinician, when cancer was more advanced or if an interpreter was required.

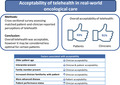

## 1 INTRODUCTION

Oncological clinical practice guidelines, both in Australia^1^ and internationally,^2^ identify the potential role of telehealth in expanding service capabilities and enabling greater equity of access to specialist cancer care, especially for people living outside of metropolitan settings. Despite this, historically telehealth has rarely been utilised in routine oncological care and there remains only limited evidence to underpin its effectiveness for addressing different components of care across the continuum.

The COVID‐19 pandemic necessitated the rapid widespread adoption of telehealth to facilitate enhanced remote healthcare access across many settings including for people with cancer.^3^ In Australia, as part of the response to the COVID‐19 pandemic, new funding models were introduced to support telehealth consultations with expanded indications.^4^, ^5^ This context afforded a rare opportunity to examine the implementation of and responses to telehealth in cancer health services on a large scale.^6^

Accordingly, there has been increasing clinical commentary on the opportunities to integrate telehealth into routine cancer practice and a particular focus on the practical considerations for effective telehealth delivery into the future.^7^ Yet, an empirical understanding of the clinical circumstances and patient populations which may be most appropriate for telehealth models remains scant.^8^, ^9^, ^10^ As such, the need to establish evidence‐based, patient‐centred models has been highlighted by cancer services,^6^ and there is a clear imperative for both patient‐ and clinician‐reported data to underpin these future models of teleoncology.

This study sought to evaluate perceptions of telehealth in real‐world oncological care through a dyadic exploration of matched patient‐ and clinician‐reported outcome data. Our primary objective was to examine patient‐ and clinician‐reported acceptability and explore individual factors which may predict greater suitability for telehealth. This was conducted with a view to understanding the ongoing applicability of telehealth and the role of individual patient, clinical and consultation characteristics underpinning a persons′ appropriateness for telehealth in future models of cancer care.

## 2 METHODS

### 2.1 Study design and setting

This study utilised a prospective, cross‐sectional, exploratory survey design to assess (matched) patient‐ and clinician‐reported perceptions of outpatient telehealth consultations occurring at a metropolitan, tertiary‐based cancer centre in Victoria, Australia. The study was guided by the checklist for good practice in the conduct and reporting of survey research described by Kelley.^11^ It was conducted as part of a broader mixed‐method study, also involving qualitative interviews with a purposive sample of patients and clinicians, the results of which will be reported elsewhere. The study received ethical approval from the St Vincent′s Hospital Human Research Ethics Committee (LRR 096/20)

The study was conducted at a time when strict public health directives were enacted including a period of 15 weeks when stringent lockdown measures were enforced in the state of Victoria, Australia. During this lockdown, there were marked limitations on leaving the house, but attendance at medical appointments was permitted.

### 2.2 Description of the telehealth model

We adopted the definition of ‘telehealth’, to include all healthcare delivered ‘at a distance’, both via video and on the telephone, reflecting a person(or patient)‐centred, as opposed to a technology‐driven approach.^12^ In‐person hospital attendance for the purposes of day administration of IV systemic anti‐cancer therapy by cancer nurse specialists, radiotherapy, examinations or admission as required continued alongside telehealth consultations, with patients separately reviewed by their medical oncologists via telehealth wherever possible even if attending for treatment. All surveillance, treatment reviews and oversight of oral treatment modalities were primarily managed with telehealth.

### 2.3 Data collection

#### 2.3.1 Procedures

All medical oncological consultations conducted by telehealth and occurring between 24 July 2020 and 11 February 2021 were identified from hospital clinics, and those patients meeting study eligibility were identified by the clinician. Patients were eligible if they were (1) receiving medical oncology cancer care at the hospital, (2) participating in a telehealth consultation and (3) able to understand spoken English without the aid of an interpreter. Patients who had participated in the study within the previous 3 months were not‐approached for subsequent inclusion.

Following completion of clinician‐reported measures at the time of telehealth consultation, patients were approached for participation within 1 week through phone or email by a member of the research team, independent from their treating team. Those who opted in were provided with the survey, administered by email or via post for those unable to complete it online. All study data were collected and managed using REDCap (Research Electronic Data Capture), a secure, web‐based software platform hosted at the University of Melbourne.^13^ Treating oncologists and patients (separately) completed the outcome measures, providing matched dyadic data through a uniquely generated code. All responses were de‐identified and personal data were not available to the researchers.

#### 2.3.2 Covariates

Demographic characteristics (patient‐reported): postcode, gender, country of birth, indigeneity, language spoken at home, regionality of home residence, relative socio‐economic disadvantage, reported in quintiles, with higher scores representing the lowest disadvantage (i.e. high socio‐economic status).^14^

Clinical characteristics (clinician‐reported): primary tumour stream, disease stage (Stages I–III/IV), performance status [Australian‐modified Karnofsky Performance Status (AKPS)],^15^ current oncological therapies.

Consultation characteristics (patient‐ and clinician‐reported): type of oncological consultation, tasks undertaken during the consultation, mode of delivery of consultation (audio ± visual), telehealth platform used, clinician cited reasons for use of telehealth, time spent during the consultation, time spent after the consultation relative to usual care, others present at the time of consultation (interpreter, family member, other health professional).

Clinician characteristics: Clinician‐reported familiarity with patient, clinician level of experience (advanced trainee vs oncologist), clinician‐rated preference for the delivery of the telehealth consultation.

#### 2.3.3 Outcome measures

Utility of telehealth: Patient perceptions of telehealth technology were assessed using the Telehealth Usability Questionnaire (TUQ), a 21‐item patient‐reported outcome measure capturing five domains of usability on a 7‐point Likert scale: usefulness, ease of use, effectiveness, reliability and satisfaction.^16^ We reported mean (SD) scores for each item and a summary score relevant to each domain, with higher values representing more favourable views. The TUQ is the psychometrically robust validated measure, with strong content validity and internal consistency.^16^, ^17^

Acceptability: Patient‐ and clinician‐reported acceptability of telehealth was (independently) assessed using a 5‐point Likert scale, where 1 = very unacceptable, 2 = unacceptable, 3 = neutral or undecided, 4 = acceptable and 5 = very acceptable. From these data, responses of 4 or 5 were collapsed to generate a binary score indicating a positive rating of ‘acceptability’.

### 2.4 Data analyses

Descriptive statistics were used to summarise the exploratory demographic, clinical and outcome variables of interest. Continuous variables were expressed as mean (SD) or median with interquartile range (IQR) and categorical variables as number (percentage). We assessed the relationship between a series of individual patient demographic, clinical, consultation and clinician characteristics with telehealth acceptability using univariate analyses reporting odds ratios, 95% confidence intervals and *p* values. Separate models were run for patient‐ and clinician‐reported outcomes. As there were multiple factors of potential significance relating to clinician views of acceptability, these were also assessed with a multivariate logistic regression model. Consistent with the exploratory aims of this study, no missing was imputed and an alpha of <0.05 was considered statistically significant. All analyses were performed using Stata version 15.1 (Stata Corp, College Station, Texas, United States of America).

## 3 RESULTS

### 3.1 Description of study population

**FIGURE 1:**
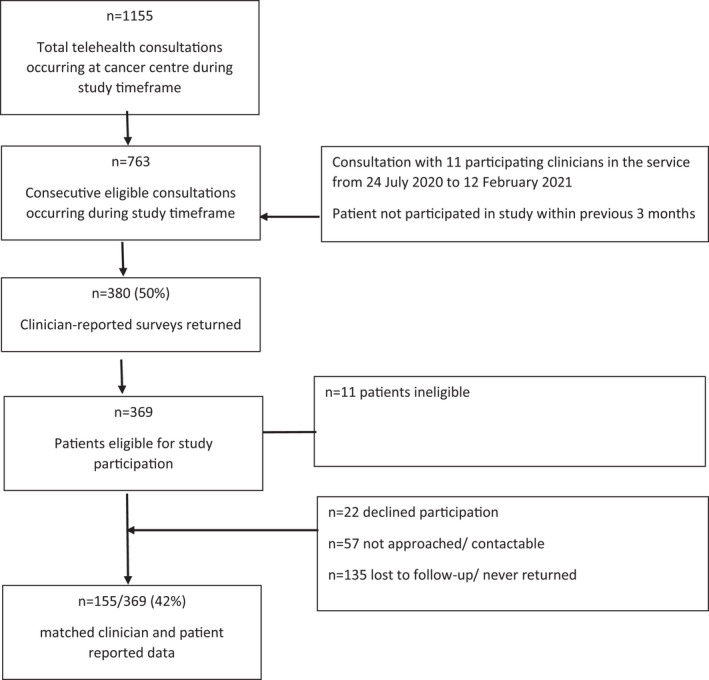
Participant flowchart

During the study timeframe, there were 1155 telehealth consultations undertaken and 763 identified as potentially eligible, representing 66% of all medical oncology consultations occurring at the cancer centre. Of these, 380 clinician‐reported surveys were completed (50% response rate), and from these, 155 (matched) patient‐reported surveys (41% response rate) were returned (Figure 1).

Patient participants included in the study population were a median age of 66 years (IQR 54, 73), predominantly female (57%), and Australian‐born (58%) (Table 1). A large minority spoke a language other than English at home (27%) and resided outside a metropolitan area (19%). Breast (32%) and gastrointestinal (27%) tumour streams were the most commonly included malignancies. Just less than half of patients had stage IV disease (46%), and 83% had a performance status above 70 indicating that they could provide self‐care but not carry out a normal activity or an active work. At the time of consultation, patients were most commonly undergoing disease surveillance (45%), receiving hormone treatment (21%) or systemic anti‐cancer therapy—either intravenously (16%) or orally (13%).

**Table 1:** Characteristics of participating patients (*n* = 155)

Demographic characteristics	
Age (years), median (IQR)	66.00 (54.00–73.00)
Gender, *n* (%)	
Male	63 (41.4%)
Female	89 (58.6%)
Australian‐born, *n* (%)	90 (58.1%)
Regionality	29 (19.1%)
Metropolitan	123 (80.9%)
Major regional cities	3 (2%)
Other regional and remote locations	26 (17.1%)
Relative socio‐economic disadvantage (SEIFA rank, Quintiles)	
1: Highest relative disadvantage (low socio‐economic status)	9 (5.9%)
2	16 (10.5%)
3	28 (18.4%)
4	44 (28.9%)
5: Lowest relative disadvantage (high socio‐economic status)	55 (36.2%)
Speaks a language other than English at home, *n* (%)	42 (27.1%)
Clinician‐reported proficiency with English	
High (e.g. Competent with English)	136 (87.7%)
Medium (e.g. English a second language, but conversant)	14 (9.0%)
Low (e.g. interpreter used or needed)	5 (3.2%)
Clinical characteristics	
Primary site of cancer, *n* (%)	
Lung	24 (15.5%)
Breast	49 (31.6%)
Gastroinstestinal	41 (26.5%)
Urogential	10 (6.5%)
Other	31 (20.0%)
Cancer stage, *n* (%)	
Stage I–III	80 (54.4%)
Stage IV	67 (45.6%)
Performance status AKPS	
0–50	7 (4.9%)
51–70	17 (11.8%)
71–100	120 (83.3%)
Current therapy, *n* (%)	
IV systemic anti‐cancer therapy	24 (15.5%)
Oral anti‐cancer therapy	20 (12.9%)
Hormone therapy	33 (21.3%)
Radiotherapy	2 (1.3%)
No anti‐cancer therapy	69 (44.5%)
Other	7 (4.5%)
Consultation characteristics	
Type of consultation, *n* (%)	
Diagnosis/first consultation	7 (4.5%)
Treatment review	68 (44.2%)
Unscheduled adverse event	2 (1.3%)
Surveillance	48 (31.2%)
Disease assessment	22 (14.3%)
Other	7 (4.5%)
Tasks undertaken during the consultation, *n* (%)	
Routine oncology	130 (83.9%)
Prognosis—good news discussion	3 (1.9%)
Prognosis—bad news discussion	4 (2.6%)
Treatment decision—new treatment	14 (9.0%)
Treatment decision—stop treatment	2 (1.3%)
Other	2 (1.3%)
Mode of telehealth delivery, *n* (%)	
Audio only (telephone)	96 (61.9%)
Audio and video	59 (38.1%)
Platform used for consultation	
Landline	25 (16.1%)
Mobile	71 (45.8%)
Health service platform (Healthdirect)	59 (38.1%)
Others present at the consultation, *n* (%)	
Interpreter	4 (2.6%)
Carer or family member	62 (40.0%)
Reason(s) for telehealth consultation, *n* (%)^a^	
Health service policy	140 (90.3%)
Patient at risk for face‐face	4 (2.6%)
Patient too ill to attend in‐person	1 (0.6%)
Patient/carer preference	38 (24.5%)
Clinician preference	49 (31.6%)
Distance from treatment	27 (17.4%)
Telehealth now standard	40 (25.8%)
Other	3 (1.9%)
Time spent during consultation in minutes, median (IQR)	15.00 (10.00–15.00)
Time spent after consultation relative to usual care, *n* (%)	
Less time	17 (11.0%)
About the same	111 (72.1%)
More time	26 (16.9%)
Clinician characteristics	
Clinician‐reported familiarity with patient	
Extremely familiar	53 (34.2%)
Moderately familiar	41 (26.5%)
Somewhat familiar	11 (7.1%)
Slightly familiar	8 (5.2%)
Not at all familiar	42 (27.1%)
Level of experience	
Consultant	122 (78.7%)
Advanced trainee	33 (21.3%)
Clinician‐rated preferred mode of delivery	
In‐person	63 (40.7%)
Telehealth—Audio only	17 (11.0%)
Telehealth—Audio + Video	74 (47.7%)

^a^ Could select more than one response.

### 3.2 Description of telehealth consultations

Most telehealth consultations occurred by telephone with audio connection only (62%), with 38% accessing video and audio through the endorsed health service platform (Table 1). The median time spent during the consultation was 15 min, with 72% of clinicians rating the time spent after the consultation as ‘about the same’ relative to usual care. In 40% of consultations, a family member or carer was also present. Among the common reasons for use of telehealth cited by clinicians included because of ‘health service policy’ (90%), rather than, for example patient preference (25%) or distance (17%) factors. The most frequent type of oncological consultations as described by clinicians were treatment reviews (44%) and surveillance (31%) and mostly categorised by tasks of routine care (84%) as opposed to a ‘breaking bad news’ type discussions such as conveying a worse prognosis (3%) or stopping treatment (1%).

### 3.3 Perceptions of telehealth

Overall, patient perceptions of telehealth were very positive (Table 2), with high mean (SD) scores reported across all domains, including usefulness, 5.81 (1.27); ease of use, 5.85 (1.35); effectiveness, 5.80 (1.41); reliability, 4.37 (1.89); and satisfaction, 5.71 (1.48). Across all domains, those who received care via the telehealth platform with a visual link to the doctor reported more favourable experiences with significantly higher mean (SD) scores compared to those who received care via the telephone limited to audio only (Table 2, *p* < 0.05).

**TABLE 2:** Perceptions of utility of telehealth (TUQ)

	All participants		Audio‐only group		Visual link group		*p* value^a^
	*N* = 155	Mean (SD)	*N* = 97	Mean (SD)	*N* = 58	Mean (SD)	
Usefulness	154	5.81 (1.27)	96	5.63 (1.37)	58	6.09 (1.03)	0.03
Telehealth improves my access to healthcare services.	148	5.52 (1.76)	91	5.25 (1.87)	57	5.95 (1.48)	0.019
Telehealth saves me time travelling to a hospital or specialist clinic.	150	6.39 (1.13)	95	6.25 (1.25)	55	6.62 (0.83)	0.056
Telehealth provides for my healthcare need.	154	5.53 (1.51)	96	5.41 (1.61)	58	5.74 (1.32)	0.18
Ease of use and learnability	152	5.85 (1.35)	95	5.61 (1.53)	57	6.24 (0.85)	0.005
It was simple to use this system.	146	6.12 (1.38)	92	5.96 (1.56)	54	6.39 (0.96)	0.068
It was easy to learn to use the system.	140	6.03 (1.50)	86	5.79 (1.67)	54	6.41 (1.09)	0.018
I believe I could become productive quickly using this system	141	5.49 (1.77)	86	5.10 (1.95)	55	6.09 (1.25)	0.001
The way I interact with this system is pleasant.	150	5.82 (1.49)	93	5.58 (1.62)	57	6.21 (1.15)	0.011
I like using the system.	151	5.58 (1.76)	94	5.31 (1.93)	57	6.04 (1.32)	0.013
The system is simple and easy to understand.	145	6.03 (1.35)	89	5.81 (1.54)	56	6.38 (0.86)	0.013
This system is able to do everything I would want it to be able to do.	149	5.16 (1.91)	92	4.79 (2.05)	57	5.75 (1.50)	0.003
Effectiveness	154	5.80 (1.41)	96	5.60 (1.57)	58	6.13 (1.00)	0.022
I can easily talk to the clinician using the telehealth system.	153	6.09 (1.45)	95	5.86 (1.62)	58	6.47 (1.01)	0.012
I can hear the clinician clearly using the telehealth system.	151	6.19 (1.25)	93	6.14 (1.31)	58	6.26 (1.16)	0.57
I felt I was able to express myself effectively.	151	5.99 (1.47)	94	5.79 (1.65)	57	6.32 (1.04)	0.032
Reliability	154	4.37 (1.89)	96	3.87 (1.89)	58	5.20 (1.57)	<0.001
I think the visits provided over the telehealth system are the same as in‐person	154	4.05 (2.11)	96	3.59 (2.09)	58	4.81 (1.93)	<0.001
Whenever I made a mistake using the system, I could recover easily and quickly.	115	5.09 (1.96)	68	4.54 (2.10)	47	5.87 (1.41)	<0.001
Satisfaction and future use	154	5.71 (1.48)	96	5.40 (1.68)	58	6.22 (0.87)	<0.001
I feel comfortable communicating with the clinician using the telehealth system.	152	5.86 (1.54)	96	5.52 (1.76)	56	6.43 (0.81)	<0.001
Telehealth is an acceptable way to receive healthcare services.	153	5.41 (1.74)	95	5.05 (1.96)	58	5.98 (1.08)	0.001
I would use telehealth services again.	152	5.90 (1.51)	95	5.63 (1.70)	57	6.35 (1.01)	0.004
Overall, I am satisfied with this telehealth system.	153	5.72 (1.66)	95	5.44 (1.84)	58	6.17 (1.17)	0.008

^a^ Scores range from 1 to7 for each question on the TUQ. Mean scores were compared between groups using a two‐sided t test.

Across all telehealth consultations, the preferred mode of delivery related to the specific interaction as rated by the treating clinician, in order to preference, was telehealth (audio + video link) 48%, in‐person (41%) and telehealth (audio only) 11%.

### 3.4 Acceptability of the telehealth consultation

Overall, patients reported high acceptability with the telehealth consultation, with 144 (93%) reporting telehealth was ‘at least somewhat’ or ‘very’ acceptable as a model of receiving their care (Table 3). Clinicians similarly reported that the telehealth consultation was acceptable for a total of 141 (91%) consultations. In general, responses between patients and clinicians were mostly concordant, whereby there were only 17 (11%) cases, where the patient and clinician assessment of the acceptability of telehealth consultation differed. Across all patient demographic, clinical, consultation‐ and clinician‐specific variables analysed (Table 4), several factors were independently associated with the clinician′s views of whether the telehealth consultation was acceptable. Clinicians were *less likely* to rate telehealth as acceptable when the patient was of older age (OR 0.93, 95% CI: 0.88–0.98, *p* = 0.005), there was an interpreter present for the consultation (OR 0.08, 95% CI: 0.01–0.62, *p* = 0.01); there was a family member present for the consultation (OR 0.16, 95% CI: 0.04–0.58, *p* = 0.006) and were *more likely* to rate telehealth as acceptable when the patient had a higher performance status (OR 1.04, 95% CI: 1.01–1.08, *p* = 0.01) and when they were more familiar with the patient (OR 6.67, 95% CI: 1.78–25.04, *p* = 0.005). Assessing these variables in a multivariate logistic regression model, only age (OR 1.06, 95% CI: 0.99–1.13, *p* = 0.05), familiarity with the patient (OR 8.20, 95% CI: 1.50–45.06, *p* = 0.02) and performance status (OR 0.04, 95% CI 1.00–1.08, *p* = 0.04) was significantly associated with clinician‐reported acceptability.

**TABLE 3:** Acceptability of telehealth consultation—patient and clinician responses (*n* = 155)

		Clinician‐reported response		
		Disagree	Agree	Total
Patient‐reported response	Disagree	2	9^a^	11 (7)
	Agree	12^a^	132	144 (93)
	Total	14 (9%)	141 (91%)	155 (100)

^a^ Discordant views.

**TABLE 4:** Univariate factors associated with patient‐ and clinician‐reported acceptability of telehealth (*n* = 155)

Predictor	Patient‐reported acceptability			Clinician‐reported acceptability				
	OR	*p* value	95% CI		OR	*p* value	95% CI	
Age*	0.975	0.307	0.929	1.023	**0.93**	**0.005**	**0.884**	**0.978**
Male	0.565	0.365	0.165	1.942	0.683	0.497	0.227	2.054
Born in Australia	1.167	0.806	0.34	4.00	1.431	0.523	0.476	4.299
Language other than English	0.627	0.476	0.174	2.264	0.64	0.45	0.202	2.034
Interpreter present*	**0.064**	**0.009**	**0.008**	**0.511**	**0.08**	**0.016**	**0.01**	**0.622**
Regional home residence	2.48	0.396	0.304	20.171	1.459	0.634	0.308	6.91
Relative socio‐economic disadvantage^a^	1.19	0.488	0.733	1.919	0.896	0.651	0.556	1.445
First telehealth appointment	2.074	0.296	0.529	8.139	0.72	0.557	0.24	2.161
Family member present*	0.786	0.702	0.229	2.698	**0.155**	**0.006**	**0.041**	**0.58**
Clinician familiarity with patient*	1.942	0.292	0.566	6.667	**6.673**	**0.005**	**1.778**	**25.041**
Audio and visual telehealth	1.086	0.899	0.304	3.885	1.607	0.442	0.48	5.383
Stage I–III disease*	**5.288**	**0.04**	**1.083**	**25.828**	0.726	0.591	0.226	2.333
Performance Status*	1.033	0.074	0.997	1.07	**1.043**	**0.01**	**1.01**	**1.078**
Routine appointment	0.5	0.518	0.061	4.091	1.475	0.574	0.38	5.72
Surveillance appointment	1.224	0.773	0.31	4.833	1.146	0.826	0.341	3.855
Time spent during consultation	0.98	0.734	0.871	1.103	0.977	0.665	0.879	1.086

Bold value indicates the statistical significance of *p* < 0.05.

^a^ SEIFA rank.

* *p <* 0.05.

There were fewer factors that independently predicted whether patients found the telehealth consultation acceptable in receiving care (Table 4). Patients were *less likely* to rate telehealth as acceptable when there was an interpreter present for the consultation (0R 0.06, 95% CI: 0.008–0.51, *p* = 0.009) and *more likely* to rate it as acceptable when they had stage I–III disease compared to stage IV (OR 5.29 95% CI: 1.08–25.82, *p* = 0.04).

## 4 DISCUSSION

This study is among the first internationally to report matched patient‐ and clinician‐reported data on the acceptability of telehealth consultations occurring in real‐world oncological care, providing novel data which bring together perspectives on telehealth both from those giving and receiving care. The results support some earlier studies suggesting that overall telehealth is perceived—by patients and their clinicians—an as acceptable mode of cancer care delivery. Building on this understanding, this study has additionally highlighted a series of factors that may be important to consider as cancer services integrate telehealth in future models of cancer care.

Our findings of matched clinician‐ and patient‐reported data revealed that while patients and their clinicians reported high overall levels of acceptability in this context and mostly concordant views, there was a minority of patients for whom delivery of care via telehealth was not preferred. We found a series of factors specific to the patient′s individual socio‐demographic and clinical situation that was associated with reduced acceptability. Those factors of the greatest importance were—from a clinician perspective: their familiarity with the patient, the patient′s age, and their performance status; and from a patient perspective: their disease stage and requirement for an interpreter.

Our findings raise several important implications for future models of telehealth, with the potential for this mode of delivery of cancer care to be considered less optimal for patients with higher complexity of need. Most notably, this included those with metastatic cancer who may have a poorer prognosis and changing health status, those who are not well known to the treating clinician, or those who identify to have additional language or technology barriers that may impede the quality of the relationship necessary for clinicians to feel confident in delivering care remotely. Future models of delivering cancer care via telehealth may benefit from considering the development of a triage process, which considers these factors, among other individual preferences for in‐person care, to determine who receives telehealth as part of routine care with perspectives of both patients and clinicians considered. These models will also require ongoing evaluation, including a much‐needed longitudinal perspective, which links patient‐ and clinician‐reported acceptability data to other safety, efficacy and health outcome data, which to date remains largely unreported.

The existing evidence around telehealth in oncological care during the pandemic, primarily limited to scoping studies including clinical commentaries and specific populations describing their perceptions of telehealth via service‐specific surveys,^8^ has broadly conveyed a less nuanced perspective of delivering cancer care via telehealth. These studies have tended to emphasise the potential benefits of telehealth, such as issues related to cost, choice and convenience,^18^ particularly when accessed by people in rural and remote areas.^19^ A study of 74 breast and gynaecological patients attending a single institution in New York reported that 92% of patients were satisfied with the use of telehealth services, with most patients indicating it saved them time (92%), and increased their access to care (73%).^20^ Another mixed cancer population attending a single institution in Houston similarly reported 92.6% were satisfied with telehealth video visits, with those who declined telehealth as an alternative to in‐person visits more likely to be older and live in lower‐income areas, and less likely to have insurance (*p* = 0.0001).^9^ Other studies again have highlighted that greater satisfaction with telehealth over physical encounters may depend on the nature of the tasks being undertaken in care, with telehealth being well suited, for example to those undergoing surveillance after active oncologic treatment.^21^

It is important to note the community context within which this study was undertaken. While in Australia, COVID‐19 case numbers have remained low, there has been heightened awareness by both patients and clinicians of the importance of minimising opportunities for community transmission including through hospital attendances. Such awareness would have influenced survey responses, and this context must therefore be considered in any future service development. A recent survey revealed that the majority (64%) of the small number of cancer physician respondents preferred in‐person visits overall and believed that virtual consultations did not provide comparable care.^10^ Similarly, there is some survey data from small cohorts of people with cancer to suggest that if given a choice, most patients (68%) prefer in‐person visits over telemedicine.^22^ Yet, other studies present contrasting views reporting most patients (76.5%) prefer video‐consulting and define it as better than or comparable to an in‐person visit.^23^ Given the heterogeneity of findings emerging, it is possible that the patient‐ and physician‐perceived threshold for balancing preference for telehealth versus in‐person consultations has and will continue to shift with the community caseload of COVID‐19 (or similar) at the time.

Despite our study providing a novel dyadic patient and clinician perspective on the acceptability of telehealth, we acknowledge several limitations which are important to consider in the planning of future studies. Although using a validated outcome measure to assess perceptions of telehealth over an exclusively purpose‐built survey, we have not assessed long‐term health outcomes in this study population, which would require many years of follow‐up. Like many existing studies, our sample may have inherent biases related to possible self‐selection for participation by those of higher socio‐economic advantage and with more positive experiences. Similarly, our results limited to patients receiving care from clinicians at a single cancer centre require external validation.

In conclusion, this study provided novel data on the acceptability of telehealth for the delivery of cancer care, bringing together the dual perspectives of patients and their clinicians. While telehealth was found to be broadly acceptable to patients and their clinicians, our results also highlighted a more nuanced set of individual factors specific to the patient′s individual socio‐demographic and clinical situation to be important. Alongside a growing heterogeneous picture emerging across the evidence base, our findings support a need to consider the patients′ level of complexity when triaging which patients may be suited to telehealth. These patient‐specific factors may be important considerations in future models of telehealth cancer services, including beyond the pandemic.

## AUTHOR CONTRIBUTION

AC was responsible for oversight of all aspects of conduct and reporting of the study. LP and AC had access to the data and OW led the analyses in consultation with AC. All authors contributed to the protocol development, clinical interpretation and write up of the results.

## CONFICT OF INTEREST

The author(s) declared no potential conflicts of interest with respect to the research, authorship and/or publication of this article.

## ETHICS STATEMENT

The study received ethical approval from the St Vincent′s Hospital Human Research Ethics Committee (LRR 096/20). All participants provided written informed consent.

## Data Availability

The data that support the findings of this study are available from the corresponding author upon reasonable request.
